# Research on the power of non-state-owned shareholders in the board and state-owned enterprises’ innovation investment

**DOI:** 10.1371/journal.pone.0341178

**Published:** 2026-02-02

**Authors:** Kanghong Li, Xiuying Wang, Yongqi Luo, Zixiao Li

**Affiliations:** 1 Research Institute of Yangtze River Culture, Yangzhou, Jiangsu, China; 2 Business School, Yangzhou University, Yangzhou, Jiangsu, China; Nanjing Audit University, CHINA

## Abstract

In the deepening stage of the reform of mixed ownership of state-owned enterprises from “mixed equity” to “reform mechanism”, how to stimulate the vitality of enterprise innovation through effective reform of corporate governance mechanism has become a core issue related to the success or failure of the reform. Existing research mostly focuses on the equity structure of non-state-owned shareholders, but generally ignores the power allocation of the board of directors, which is a key link in transforming equity advantages into governance efficiency. Based on the perspective of board functions, this paper discusses the impact and mechanism of the board of directors’ board power on the innovation investment of state-owned enterprises from 2008 to 2022. The research finds that the power of non-state-owned shareholders in the board has a significant promotion effect on SOEs’ innovation investment. The results of the robustness test show that such a finding holds. According to mechanism analysis, the interplay intensity in the board serves as sort of mediator for the impacts of the power of non-state-owned shareholders in the board on SOEs’ innovation investment. The power of state-owned shareholders in the board plays an inverted U-shaped role in the positive promotion exerted by the power of non-state-owned shareholders in the board on SOEs’ innovation investment, that is, along with the expansion of the power of state-owned shareholders in the board, the positive impacts of the power of non-state-owned shareholders in the board on innovation investment are reinforced before being inhibited.

## Introduction

According to J·A Schumpeter’s theory of innovation, innovation involves the recombination of production factors and conditions, essentially establishing a new production function that generates value [1]. It follows that innovation investment is the important precondition on which the enterprise creates value and achieves high-quality development. According to the *China R&D Expenditure Report 2024*, China’s total national R&D expenditure has exceeded 3.6 trillion yuan, with an R&D intensity of 2.68%. Although the growth rate has moderated compared to 2023, there remains significant room for improvement in R&D investment [2]. In terms of the entities performing R&D activities, China’s execution structure has gradually shifted from a “dual-core system of enterprises and research institutions” to an “enterprise-dominated model.” In nature, SOEs undertake both economic and social responsibilities. Given such nature and the conditions restricting their functional positioning, they face dual challenges: while pursuing economic efficiency, they must also shoulder substantial policy burdens and strategic missions [3]. On the other hand, SOEs inherently suffer from owner absence and insider control issues. Management tends to prioritize self-interest maximization, exhibiting risk-averse decision-making patterns [4], thereby reducing investments in innovation activities. Empirical studies also provide supporting evidence for this. It has been found that, based on data from Chinese listed companies, the average R&D intensity of SOEs is significantly lower than that of non-state-owned enterprises.[5] Additionally, scholars have verified that both state control and state ownership exert a significantly negative impact on corporate innovation performance [6]. In a rapidly changing external environment, clarifying how to stimulate SOEs’ intrinsic innovation motivation and maintain sustainable competitive advantage has become an issue of significant theoretical value and urgent practical importance [7].

The key of deepening the reform of SOEs on all fronts lies in advancing the mixed ownership reform of SOEs. Advancing mixed-ownership reform has become a critical breakthrough. Non-state-owned shareholders possess flexible market management philosophies, forward-looking market awareness, advanced technological knowledge, and stronger innovation capabilities. They are better positioned to identify policy opportunities and market prospects, and can transmit such insights to state-owned shareholders. Meanwhile, state-owned shareholders leverage political resources and substantial assets to achieve multi-dimensional complementary advantages through resource spillover and integration effects, thereby enhancing innovation investment [8]. Consequently, the introduction of non-state-owned equity optimizes both the ownership structure and decision-making quality of SOEs [9], while alleviating the pressure on any single major shareholder to bear innovation risks alone. Existing studies have primarily examined the governance role of non-state-owned shareholders from two dimensions: ownership structure and top-level governance [10–12]. However, existing research indicates that merely introducing non-state-owned shareholders is insufficient to fully realize the governance effects of non-state capital [13]. In corporate management, the board of directors serves as a critical decision-making and monitoring body [14], exerting substantial influence on innovation-related decisions. Therefore, granting non-state-owned shareholders meaningful board voice allows them to actively contribute to operational goal-setting and resource allocation. This participation is essential for SOEs to effectively address issues such as “absence of owners,” extended agency chains, and insider control [15,16]. This enhanced participation strengthens oversight over state-owned shareholders and management, facilitates the effective transmission of innovation-related information, and ultimately promotes increased innovation investment. In practice, the appointment of directors by non-state-owned shareholders has emerged as a defining feature of deepening mixed-ownership reform. As of 2021, more than 80 out of the 208 SOEs that had launched the mixed ownership reform had granted certain board power to non-state-owned shareholders so as to adjust and improve their corporate governance structure and mechanism. This practical trend aligns closely with the “board-centric” reform emphasized in the new *Company Law of the People’s Republic of China* that took effect on July 1, 2024. The revised legislation reinforces the board’s central role in corporate decision-making, providing non-state shareholders with new legal grounds and operational space to participate in governance through director appointments. Accordingly, this paper examines how the power of non-state shareholders on the board affects corporate innovation investment. It employs an integrated analytical framework that incorporates mixed-ownership reform, board governance, and corporate innovation investment. Mixed-ownership reform extends beyond mere changes in equity structure; at a deeper level, it reshapes the board structure and internal power configuration of state-owned enterprises by introducing non-state shareholders and their appointed directors. This reallocation of governance power helps mitigate the inherent agency problems in SOEs, enabling the board to better fulfill its strategic decision-making and oversight functions. Consequently, it influences both the decision-making process and the level of investment in long-term, high-risk strategic resources such as innovation.

Current research on innovation investment is fragmented in its analysis of internal and external influencing factors. Internal elements include equity structure, board governance, and corporate strategy [17,18], while external factors encompass economic policy uncertainty and market-oriented reforms [19,20]. Literature on board power lacks clear definitions, with studies on non-state-owned shareholders ‘influence remaining superficial in power distribution analysis. Most existing research adopts a governance participation perspective [21,22], treating equity structure and board power as separate variables. These studies examine their impacts on corporate behavior without distinguishing their mechanisms. Although existing literature has addressed the role of non-state-owned shareholders’ equity ratios in state-owned enterprises [23,24], few investigations explore the practical implications of non-state-owned directors’ actual participation in governance decision-making processes. While some scholars have examined the board level [25,26], few have explored the operational mechanisms behind over-appointed directors. To bridge this theoretical and practical gap, this paper clarifies the impacts of the power of non-state-owned shareholders in the board on a SOE’s innovation and the relevant action mechanism to ensure that mixed ownership reform can produce the desired effect is what the realities call for and what this paper focuses on. The paper comes up with the following three research breakthroughs.

First, this paper shifts the focus of its contribution from the mere presence of non-state directors to how board power is allocated, thereby highlighting the governance implications of internal board power structures. Although prior studies have examined the existence of non-state directors and their general governance roles, most have not sufficiently explored the relative allocation and structural configuration of power within the board. This paper focuses on a critical governance context characterized by an asymmetric allocation of control rights and cash-flow rights arising from the over-appointment of directors by non-state shareholders. By doing so, it not only examines the governance effects of non-state shareholders’ board power, but also clarifies how the relative strength and structural imbalance between non-state and state shareholders reshape firms’ strategic decision-making space. This perspective goes beyond existing studies that approach mixed-ownership reform primarily from ownership structure or macro-level governance dimensions [21,12], and provides a novel theoretical lens for understanding how mixed-ownership reform influences firms’ long-term strategies through internal power reconfiguration.

Second, this paper uncovers the transmission mechanism of “board power-board functions-firm innovation” thereby opening the black box of the governance process. While existing research has extensively investigated internal and external determinants of innovation investment [27,28], limited attention has been paid to the board-level governance processes underlying these effects. This paper constructs and empirically validates a transmission pathway linking board power, board functions, and firm innovation. Specifically, it shows that non-state directors enhance the board’s strategic advisory capacity and monitoring effectiveness by increasing board meeting frequency, thereby transforming the allocation of governance power into tangible impacts on the allocation of innovation resources. This mechanism-based analysis helps bridge the gap in the literature between board behavioral processes and firms’ innovation decisions.

Third, this paper clarifies the boundary conditions of non-state shareholders’ governance effectiveness and provides micro-level evidence from a power-interaction perspective to inform the differentiated advancement of state-owned enterprise reform. Within the institutional context of China’s mixed-ownership reform, this paper systematically examines the moderating roles of state shareholders’ board power, SOE hierarchical level, enterprise category, and industry heterogeneity. The findings identify the conditions under which the influence of non-state shareholders on innovation investment is strengthened or weakened, thereby deepening our understanding of the boundaries and contingency factors shaping their governance effectiveness. These results offer richer theoretical explanations and policy implications for advancing SOE reform in a classified and hierarchical manner and for promoting firm innovation through the optimization of board power structures.

## Literature review

### Relevant research on the relationship between the power of non-state-owned shareholders in the board and SOE governance

The board of directors, the core of corporate governance, play important roles in decision-making, consultation, supervision and power balancing [29], which all have direct influence on corporate behaviors [30]. The existing research on board power focuses on the structural power of the board and the say in the board [31]. The differentiated distribution of organizational structures and hierarchical personnel, such as director post and salary, exerts different impacts on the decision-making of the organization [32]. The say in the board is contingent upon the shareholders’ holding proportion and the possessed seats in the board [33]. Since the initial reforms in 1978, SOEs have undergone multiple evolutionary phases—from decentralization and profit retention to institutional innovation, and further to systemic restructuring and categorized reforms [34]. Throughout this process, actively developing a mixed-ownership economy and advancing SOE mixed-ownership reform have emerged as critical pathways for achieving high-quality deepening of reforms. Moreover, the new *Company Law of the People’s Republic of China* marks a governance shift from “shareholder-centricism” to “board-centricism,” granting boards greater autonomy over corporate operational decisions and reinforcing the central role of both the board and individual directors in corporate management and strategic oversight. In this paper, SOEs under the mixed ownership serve as the research target. The alteration of board power is mainly attributed to the alteration of the equity structure of the enterprise once non-stated capital is introduced. In line with The Company Law and corporate articles and regulations, shareholders can nominate or recommend several directors through the board of shareholders. The board of directors observes the one-person-one-vote practice, and each director is entitled with such voting power. In this paper, the board power refers to the say of a director when board voting takes place and is measured by the number of seats obtained in the board. Given the equal and asymmetric allocation between equity and control rights, research on the power of non-state-owned shareholders in the board mainly proceeds at two levels, namely, appointing directors and over-appointing directors [35].

### Board power parity and SOE governance

Research on non-state-owned shareholders’ participation in corporate governance provides a foundation for examining their power on the board, given the principle of aligning control rights with equity. In the early stage of SOE mixed-ownership reform, scholarly attention centered primarily on the equity structure and its collaborative governance effects. According to Xia [36], to SOEs under the mixed ownership reform with diversified equity, the differences in mixed scale and degree lead to varied impacts upon corporate capital costs. Yu et al. [37] studied the impacts of non-state-owned shareholders on corporate social responsibility and economic performance in terms of shareholder diversity, shareholders’ penetration degree and degree of mixed equity (DME). However, as the SOE mixed ownership reform deepens, practical experience shows that focusing solely on equity structure may have limitations. Mere “mixing of equity” does not necessarily translate into “mechanism reform.” Zeng et al. [38] found that non-state-owned shareholders’ merely holding shares does not produce the expected positive effect on accounting information quality while appointing senior executives can significantly improve the accounting information quality of SOEs. Hence, many studies began to discuss the impacts of non-state-owned shareholders’ participation in governance from the perspectives of equity structure and senior executive governance [21,22]. Senior executive governance refers to the power in the board obtained by non-state-owned shareholders, given the equal allocation between equity and control rights. In terms of the effect of SOE governance, that non-state-owned shareholders appoint directors can produce the desired effects in management and decision-making such as removing overcapacity, improving asset allocation efficiency [39], addressing the de-zombification problem [40], ensuring state-owned asset preservation and appreciation [41] in addition to moderating the principal-agent issue and improving accounting information governance [38,42].

In summary, there has been rich research on the relationship between board power and SOE governance in light of the equal allocation between equity and control rights, establishing board power as the core governance channel. However, in both practice and theory, even when non-state shareholders obtain equivalent board power, their governance effectiveness still faces challenges. This has prompted scholars to focus on deeper issues of power distribution.

### Excess board power and SOE governance

Despite the positive governance effects of non-state-owned shareholders after equal power proved by substantial research, some researchers found that the mixed ownership reform has not yet achieved the desired effect after non-state-owned shareholders possess the power in the board. Ma and Zhang argued [43], although non-state-owned directors who tend to be short-sighted can promote groundbreaking innovation, they significantly inhibit exploratory innovation that is fund-consuming and risky. Du [44] found that, when the equivalence between equity and control rights of non-state-owned shareholders is increased, the phenomenon of non-state-owned shareholders’ pursuing immediate interests can be reduced, in other words, the over appointment of directors by non-state-owned shareholders can inhibit the self-interest tendency of non-state-owned directors. In realities, the phenomenon that non-state-owned shareholders over appoint directors to participate in SOE governance has attracted increasing attention. Take China Unicom that has completed its mixed ownership reform as an example, four out of its eight seats in its board of directors were appointed by strategic investors, thus creating a situation in which board powers are mutually checked and balanced against between state-owned directors and non-state-owned directors. This logic is supported by Liu et al. [45] whose research targeted central state-owned enterprises under mixed ownership. The quantity of research on over appointment of directors remains small. Taking major shareholders’ opportunist behaviors as the starting point, Zheng et al. [46] studied the negative impacts of actual controllers’ excess board power on the supervisory function of the board of directors. However, Li et al. [47] maintained that actual controllers’ over appointing directors can improve the decision-making efficiency of the board and stimulate the enterprise to launch innovation initiatives. In a SOE under the mixed ownership reform, usually state-owned shareholders remain the actual controllers. Non-state-owned shareholders, as latecomers, tend to have a relatively low shareholding proportion. As non-controlling shareholders, non-state-owned shareholders cannot make up for their weak voice in the board unless they resort to the approach of over appointing directors [48]. Feng and Guo [35] heeded the inferiority of non-state-owned shareholders, tested and proved that non-state-owned shareholders’ over appointing directors can significantly improve the quality of accounting information. In summary, the impact of non-state shareholders’ over-appointment on SOE governance remains a complex and underexplored area, presenting a ‘black box’ worthy of further investigation.

### Research hypotheses

#### The relationship between the power of non-state-owned shareholders in the board and SOEs’ innovation investment.

Unlike in non-state-owned enterprises, corporate governance in SOEs places particular emphasis on the board of directors. Enhancing board governance effectiveness has therefore become a pivotal issue in improving SOE governance mechanisms. The “absence of owner” and the incomplete manager system reform among SOEs made directors or board of directors the important party in the principal-agent relationship, testified to by the “Chinese-styled control by insiders’ problem [49]. The governance of SOEs, unlike that of non-SOEs, prioritizes the board of directors. How to make board governance more efficient is the key to improving the SOE governance mechanism. In addition, in the practice of SOE mixed ownership reform, how to coordinate control rights of an enterprise is of particular importance [50]. As the agent of shareholders, the board exercises rights on behalf of respective parties when performing its supervisory or decision-making functions. Therefore, in the course of deepening the reform of the mixed ownership, it is important to take into account the different nature of shareholders and the allocation of power formed by the directors appointed by such shareholders, and on this basis explore the improvement of the effectiveness of board governance so as to influence an enterprise’ innovation behaviors [28].

In terms of the supervisory function of the board, non-state-owned shareholders can strengthen the supervision of the “political promotion motivation” and “risk aversion tendency” of SOEs’ senior executives by over appointing directors, so as to improve the level of innovation investment of enterprises. On one hand, influenced by administrative hierarchies, SOE directors often exhibit strong political career incentives [51,52]. This may lead to inefficient resource allocation, as described in the phenomenon of “spending others’ money without regard for efficiency” or “using others’ resources for personal objectives” [49]. Such behavior can constrain the enterprise’s financial capacity to undertake value-creating initiatives. In contrast, non-state shareholders are profit-driven investors for whom “protecting the purse” forms the foundation of capital accumulation and expanded reproduction. As representatives of these shareholders, non-state directors appointed to the board actively fulfill their duties by curbing activities in which state-affiliated directors or executives pursue private benefits at the expense of innovation investment. On the other hand, innovation constitutes a long-term, creative endeavor characterized by significant uncertainty and risk. To fulfill political mandates and maintain socio-economic stability, state-owned major shareholders may deviate from profit-maximization objectives and adopt more conservative operational models [52,53], thereby reducing investment in high-risk activities. In contrast, non-state directors, representing the interests of capital providers, prioritize long-term development and value appreciation. They view innovation as a vital pathway to enhance corporate long-term value and achieve capital appreciation [54], and therefore actively monitor and counterbalance decisions by state-affiliated directors and executives.

In terms of the decision-making function of the board, non-state-owned shareholders’ appointing directors is beneficial to improving the efficiency and quality of decision-making of the board, thus driving SOEs to increase their investment in innovation. Firstly, innovation capacity is a function of an enterprise’s ability to acquire and integrate new knowledge. Leveraging the resources and platforms of SOEs, non-state directors excel in building high-level commercial and technological relational capital. This enables them to transform external innovation opportunities into internal innovation activities at lower costs, leading to more targeted and efficient R&D investments [55]. Furthermore, the decision-making mindset of non-state directors gradually influences that of their state-appointed counterparts, leading traditionally “administrative-oriented” directors to adopt more “economically-driven” perspectives. Secondly, according to behavioral decision theory, the complex and information-asymmetric nature of innovation environments often lead to reduced risk tolerance when decision-making power is decentralized. The inclusion of non-state directors helps establish a balanced power structure among multiple major shareholders, mitigating the dominance of state-owned controlling shareholders or insiders. This fosters a collective decision-making process involving diverse interest groups [56]. Consequently, innovation investment decisions become outcomes of negotiation and compromise between non-state-appointed directors and other board members.

However, the first condition for making the power of non-state-owned shareholders in the board capable of improving the governance effectiveness of the board is that non-state-owned shareholders can have a substantial say in the board. Even by appointing directors to enter the board of a SOE, non-state-owned shareholders may not necessarily have their voice heard in the board. In the governance culture observing a strict rule of status and seniority, whether the weak non-state-owned directors can have a say is subject to the chairperson of the board who holds the authority. In a governance culture drawing a clear line between insiders and outsiders, non-state-owned directors are considered as outsiders and they may find it difficult to perform their functions of decision-making and supervision [35,57]. When control rights exceed equity ownership, the effect of non-state shareholders on innovation investment becomes stronger and more pronounced. Therefore, non-state-owned shareholders can more effectively grasp the right to speak by over-appointing directors [46]. Over appointing directors break the equal allocation of “one share, one vote”, granting non-state-owned shareholders a decision-making influence disproportionate to their shareholding ratio [45]. The power of non-state-owned shareholders in the board of directors significantly increases, which helps non-state-owned directors better play the roles of “supervisor” and “decision-maker”. Drawing on resource dependence theory, non-state shareholders serve as resource providers, supplying SOEs with critical assets such as long-term strategic vision and flexible decision-making mechanisms [58]. Consequently, over appointments increase both the frequency and support for innovation-related discussions during board deliberations, strengthening the substantive voice of non-state shareholders. Furthermore, this governance arrangement enhances the priority and stability of R&D budgets in internal resource allocation processes [59], increasing investment in innovation. Based on the above, this paper brings forward the following hypothesis:

**H1**: the power of non-state-owned shareholders in the board can boost SOEs’ investment in innovation, in other words, both non-state-owned shareholders’ appointing directors and over appointing directors can significantly make SOEs amplify their investment in innovation.

#### The mediating role of board meeting frequency in the board.

A chief means for directors to participate in governance is to attend the board meeting [60], where major events including enterprises’ innovation will be voted on. Strong interplay in the board is the key to the effective governance of the board, for it mirrors that the board actively performs its functions [61]. If the number of board meetings is too high, the decision-making efficiency of the board is considered low, and the enterprise’ s fiscal performance will be negative [62]. However, this is not the major cause of low efficiency of decision-making for SOEs. The real cause is the chairperson’s arbitrary style, which makes board meetings fall victim to formalities [63]. When non-state shareholders obtain board power and appoint directors, the resultant increase in meeting frequency is likely to be accompanied by more substantive governance activities. Directors appointed by non-state shareholders can bring external market perspectives and exhibit greater initiative in active oversight. Frequent board meetings generate higher-quality monitoring and advisory effects [64], improve information exchange and strategic deliberation, and thereby promote innovation investment. The solution, therefore, will be that non-state-owned directors effectively check and balance against state-owned directors by intensifying the interplay in the board so as to improve the decision-making quality of the board.

First, the board power of non-state-owned shareholders significantly increases the demand for board interaction. When non-state-owned shareholders appoint directors to SOE boards, they create a heterogeneous governance structure. This structure enhances interaction intensity through strengthened monitoring functions and improved information environments. Non-state-owned directors typically bring market-oriented experience and technical expertise. To thoroughly assess the investment feasibility and potential risks of innovation projects, they often require more frequent board meetings and specialized discussions [33]. From the perspective of asymmetric information, state-owned directors have long held the “insider advantage” within SOEs [64], controlling critical information such as technology development and fund utilization. In contrast, non-state-owned directors, as “external participants,” need to attend board meetings more frequently [61]. This allows them greater opportunity to access corporate information and make informed decisions during board voting. Furthermore, the participation of non-state-owned directors breaks the tradition of monolithic decision-making in boardrooms. When divergent viewpoints emerge, non-state-owned directors require more extensive deliberation to effectively fulfill their monitoring and decision-making responsibilities. This inevitably increases the frequency of substantive interactions during board meetings [65].

Second, enhanced board interaction intensity promotes innovation investment. According to Resource Dependence Theory, non-state-owned shareholders can provide SOEs with technological resources, market channels, and R&D networks [66]. Increased board interaction intensity implies more frequent information exchange within the board [67], which mitigates information asymmetry. This enables non-state-owned directors to better monitor the use of innovation resources [28] and helps prevent state-owned directors from diverting resources to non-productive areas for private gain [68]. In addition, in the course, state-owned directors gain access to more external information. For example, non-state-owned directors’ consideration affects, to some extent, the decision-making logic of the board, and thus lowers the possibility that state-owned directors refuse to increase investment in innovation for personal benefit. Therefore, the power of non-state-owned directors in the board effectively checks and balances against state-owned directors by intensifying the interplay in the board and improving the monitoring function and decision-making quality of the board. It will encourage SOEs to increase their investment in innovation. Based on the above, this paper brings forward the following hypothesis:

**H2:** the power of non-state-owned shareholders in the board can propel SOEs to invest in innovation by increasing board meeting frequency.

#### The moderating role of the power of state-owned shareholders in the board.

On the topic of the power in the board, most researchers only considered the power of one party but overlook that of other parties. Take Li and Yi [69], they found that non-actual controllers’ say in the board can positively promote SOEs’ innovation. In contrast, Li et al. [70] tested and proved that actual controllers’ over appointing directors can stimulate the enterprise to launch innovation initiatives. The above indicates that either actual controllers or non-actual controllers will influence an enterprise’s innovation initiatives once they possess power in the board. When the power of non-state-owned shareholders in the board is studied, examining the power of non-state shareholders in isolation is inadequate, as this approach overlooks the interactive effects of power distribution among different shareholder groups. Instead, both non-state-owned shareholders and state-owned shareholders should be considered simultaneously. Board power allocation is inherently complex, involving competition and balance among multiple interest groups. To address this gap, this paper introduces the power of state-owned shareholders on the board as a moderating variable, investigating its impact on the relationship between the power of non-state-owned shareholders in the board and a SOE’s investment in innovation.

As mentioned above, compared to non-state-owned directors, state-owned directors have risk aversion. The decision-making logic of non-state-owned directors is strategic, for it aims at the long-term development of the enterprise to increase investment return. In this sense, the power of non-state-owned shareholders in the board can advance the enterprise’s investment in innovation. For one thing, in the board, non-state-owned shareholders can form the force checking and balancing against state-owned shareholders. Especially, when non-state-owned shareholders over appoint directors, the short-sighted behaviors of state-owned shareholders can be effectively inhibited. For another thing, the two parties have their own interests to consider. Inevitably, there are heated discussions before the board vote on important matters. So, the board can be urged to actively perform their functions so as to improve their governance effectiveness. When the power of state-owned shareholders in the board is appropriately checked and balanced against, SOEs’ natural policy superiority can be sustained, and the restrictions on the enterprise’s financing can be relaxed so as to promote investment in innovation [71]. Furthermore, under the catfish effect, non-state-owned directors are spurred to actively perform their functions. In this case, the two parties mutually check and balance against each other, and state-owned and non-state-owned capital can achieve mutual complementarity in the interest of common development.

In the board, state-owned shareholders and non-state-owned shareholders are opponents to each other, and to some extent the size of the power in the board is in a trade-off between the two parties. When the power of state-owned shareholders in the board is extremely large, non-state-owned shareholders cannot achieve balance with state-owned shareholders in the board even if the former obtain excess seats in the board. In that case, the power of state-owned shareholders is beyond the tolerable degree. Even if non-state-owned shareholders can voice their opinions in the board, their opinions cannot produce the desired effect. As a consequence, non -state-owned shareholders become less motivated to participate in decision-making and supervision [28]. The effect of the power of non-state-owned shareholders in the board on the innovation investment gets dented. When non-state-owned shareholders obtain corresponding and even excess seats in the board, their say in the board will be greatly improved. They can gain access to more inside information, exert greater impacts on the decision-making of the board. However, it may also spawn a short-sighted mindset among them. They have to make a choice between “return on innovation” and “short-term profit” [43,44]. Based on the above, only when state-owned shareholders are appropriately checked and balanced against can the positive effect of the power of non-state-owned shareholders in the board work. Based on the above, this paper brings forward the following hypothesis:

**H3:** The power of state-owned shareholders in the board plays an inverted U-shaped role in the positive promotion of the power of non-state-owned shareholders in the board on the innovation investment of state-owned enterprises.

## Research design

### Data sources

In this paper, the sampling periods range from 2008 to 2022, and the research targets are A-shares state-owned listed companies on Shanghai Stock Exchange (SSE) and Shenzhen Stock Exchange (SZSE). Referring to the research by Feng et al. [72] and Li et al. [28], the authors of this paper take the year 2008, the critical time of the equity division reform, as the starting point of the sampling periods. The raw samples were processed in the following ways. (1) The samples of ST and *ST were removed; (2) Samples with incomplete data for each variable were removed; (3) Samples from the financial industry were removed; (4) To avoid the impacts of extreme values, the main variables were winsorized at the 1st and 99th percentiles. Data of actual controller nature, share holding ratio, director appointing and relevant fiscal data came from China Stock Market & Accounting Research Database (CSMAR) and Chinese Research Data Services Platform (CNRDS). The missing data of this paper, such as R&D investment and non-state shareholders’ appointing directors, were obtained by manually reviewing the R&D expenses or investment, the information of top ten shareholders, and the SOE’s directors’ appointment in a non-SOE from the listed SOE’s annual reports. The final number of samples reached 7,746. The data of this paper were processed by STATA16.0.

### Definition of variables

#### Definition of variables.

Explained variable: Innovation investment (Rd)

Innovation investment refers to the total sum of production factors invested in innovation initiatives [73]. It is a straightforward indicator of the changes in the enterprise’s intentions and actions of innovation. In this paper, relative indices were employed to measure innovation investment. Referring to the research by Cheng and Chen [10] and Ren et al. [11], the authors of these papers used the ratio of the enterprise’s R& D investment to revenues to measure a SOE’s innovation investment level.

Explanatory variable: the power of non-state-owned shareholders in the board (N_directorratio,N_gap).

With reference to the research by Feng and Guo [35], Li and Li [33], this paper measured the power of non-state-owned shareholders in the board with two indices from the perspectives of appointing directors and over appointing directors. (1) The ratio of non-state-owned shareholders’ appointing directors (N_directorratio). The index is the number of directors appointed by non-state shareholders divided by the number of non-independent directors in the board. (2) The ratio of non-state-owned shareholders’ appointing excess directors (N_gap). This index is the difference between the ratio of directors appointed by non-state shareholders and the ratio of shares held by non-state-owned shareholders.

Mediator: board meeting frequency (Meeting)

Experienced directors can offer more resources and information relevant to decision-making [74] and intensify the supervision among directors in the board [75]. As the number of meetings conducted by the board increases, directors have more opportunities to actively participate in decision-making. In turn, they have more opportunities to perform their supervisory function [62].

Moderator: the power of state-owned shareholders in the board (S_directorratio)

The definition and the measuring method of this variable are the same as those of the ratio of directors appointed by non-state-owned shareholders in “the power of non-state-owned shareholders in the board”. Drawing on the studies by Zheng et al. [46], Feng and Guo [35], the ratio of directors appointed by state-owned shareholders (S_directorratio) is the number of directors appointed by state-owned shareholders divided by the number of non-independent directors in the board.

#### Control variables.

With reference to Feng and Guo [35], Zheng et al. [46], relevant control variables were selected. At the same time, the impacts of the variables of Industry and Year were considered. See Table 1 for details.

**Table 1:** Variable description.

Type	Title	Code	Measuring way
DV	Innovation investment	Rd	R & D investment divided by revenues
		N_directorratio	Number of directors appointed by non-state-owned shareholders/(board size-independent director number)
		N_gap	Difference between ratio of non-state-owned shareholders’ appointing directors and ratio of non-state-owned shareholders’ holding
Mediator	Board meeting frequency	Meeting	Number of board meetings
Moderator	Power of state-owned shareholders in the board	S_directorratio	Number of directors appointed by state-owned shareholders/(board size-independent director number)
Control variable	Asset-liability ratio	Lev	Overall liabilities divided by overall assets
	Equity concentration	Top1	Ratio of the largest shareholders’ holding
	Enterprise age	Age	In (Sample observation year-company founding year+1)
	Enterprise size	Size	Take the natural logarithm of total assets
	Dual posts as both the chairperson & the general manager	Dual	1 if the chairperson and the general manager are the same person; otherwise, 0.
	Independent director ratio	Independentratio	Ratio of independent directors in the board of directors
	Enterprise growth	Growth	Main business income growth rate
	Overall return on assets	Roa	Net profit divided by average total assets
	Board size	Boardsize	ln(number of board members)
	Industry	Industry	Dummy variables are assigned based on the industry codes in the “Guidelines on Industry Classification of Listed Companies” revised by the China Securities Regulatory Commission in 2012.
	Year	Year	Dummy variable; the sample interval of this study is from 2008 to 2022.

### Model design

To study the impacts of the power of non-state-owned shareholders in the board on SOEs’ investment in innovation, four empirical regression models were designed, including the model of the mediator effect and that of the moderator effect.


$${\text{Rd}}_{\text{i},\text{t}}=\alpha +\beta_{\text{1}}{N\_directorratio}_{i,t}/{N\_gap}_{i,t}+\beta_{\text{2}}{\sum Controls}_{i,t}+\epsilon_{\text{i},\text{t}\;}$$ (1)

$${\text{Meeting}}_{\text{i},\text{t}}=\alpha +\beta_{\text{1}}{N\_directorratio}_{i,t}/{N\_gap}_{i,t}+{\beta_{\text{2}}\sum Controls}_{i,t}+\epsilon_{\text{i},\text{t}\;}$$ (2)

$${\text{Rd}}_{\text{i},\text{t}}=\alpha +\beta_{\text{1}}{N\_directorratio}_{i,t}/{N\_gap}_{i,t}+\beta_{\text{2}}{Meeting}_{i,t}+\beta_{\text{3}}{\sum Controls}_{i,t}+\epsilon_{\text{i},\text{t}\;}$$ (3)

$$\begin{array}{cc} \;{\text{Rd}}_{\text{i},\text{t}}= & \alpha +\beta_{\text{1}}{N\_directorratio}_{i,t}/{N\_gap}_{i,t}+\beta_{\text{2}}{S\_directorratio}_{i,t}+\beta_{\text{3}}{{S\_directorratio}^{\text{2}}}_{i,t}\\ & {+\beta }_{\text{4}}{{N\_directorratio}_{i,t}/{N\_gap}_{i,t}*S\_directorratio}_{i,t}{+\beta }_{\text{5}}{{N\_directorratio}_{i,t}/{N\_gap}_{i,t}*{S\_directorratio}^{\text{2}}}_{i,t}\\ & {+\beta_{\text{6}}\sum Controls}_{i,t}+\epsilon_{\text{i},\text{t}\;} \end{array}$$ (4)

The explained variable Rd denotes the innovation investment level of SOEs. The explanatory variable denotes the power of non-state-owned shareholders in the board, measured by two variables, namely, N_directorratio and N_gap. The mediator Meeting denotes the intensity of interplay in the board. The moderator, the power of state-owned shareholders in the board, denoted as S_directorratio, was measured by the ratio of directors appointed by state-owned shareholders. Controls denote control variables, α denotes the intercept, βi denotes the coefficient, and ε denotes the residual.

## Empirical results and analysis

### Descriptive statistics

This paper conducted descriptive statistics on the main variables, with the results shown in Table 2. Data show that from 2008 to 2022, the average ratio of R&D investment to operating revenues (Rd) for state-owned enterprises was 2.82%, significantly lower than that of listed companies during the same period (4.75%). The median of innovation investment was 2.1%, and the maximum was 17.7%, indicating that the level of innovation investment by listed state-owned enterprises in China was relatively low. Of the state-owned enterprises under mixed ownership, the average ratio of appointed directors (N_directorratio) being 5.46% the average ratio of excess appointed directors (N_gap) being −7.15%. This indicates that of the state-owned enterprises under mixed-ownership, there are still relatively few enterprises in which non-state-owned shareholders obtain power in the board, and the power of non-state-owned shareholders in the board remains relatively small. In terms of the power in the board of a SOE, the average proportion of directors appointed by state-owned shareholders (S_directorratio) was 38.34%. With the power of non-state-owned shareholders in the board taken into account, state-owned shareholders still possess a relatively large say in the board.

**Table 2:** Variable descriptive statistics.

Variable	Sample size	Mean	SD	Min.	Median	Max.
Rd	7746	0.0282	0.0310	0.0000	0.0210	0.1777
N_directorratio	7746	0.0546	0.1332	0.0000	0.0000	0.6667
N_gap	7746	−0.0715	0.1295	−0.4766	−0.0562	0.3899
Meeting	7746	9.6863	3.8099	4.0000	9.0000	24.0000
S_directorratio	7746	0.3834	0.2884	0.0000	0.3485	1.0000
Lev	7746	0.5049	0.1925	0.0862	0.5146	0.9244
Top1	7746	0.3815	0.1478	0.1106	0.3680	0.75.46
Age	7746	2.7145	0.4424	1.3863	2.8332	3.4012
Size	7746	22.9294	1.4191	20.2000	22.7600	27.1000
Dual	7746	0.0909	0.2875	0.0000	0.0000	1.0000
Independentratio	7746	0.3721	0.0559	0.3077	0.3333	0.5714
Growth	7746	0.3363	0.8241	−0.6369	0.1286	5.7912
Roa	7746	0.0457	0.0541	−0.1665	0.0428	0.2190
Board	7746	2.3092	0.1746	1.3863	2.3026	2.9444

### Empirical results

#### The relationship between the power of non-state-owned shareholders in the board and SOEs’ innovation investment.

This study conducted multiple regression analysis on the relevant variables. The regression results are shown in Table 3. The results show that the regression coefficients of the ratio of appointed directors (N_directorratio) are positively significant at the 1% level, indicating that the appointment of directors by non-state-owned shareholders significantly boosts the innovation investment of state-owned enterprises. The regression coefficients of the ratio of appointing excess directors (N_gap) are positively significant at the 1% level, indicating that the excess power obtained by non-state-owned shareholders in the board also contributes to the increase in the innovation investment of state-owned enterprises. Both non-state-owned shareholders’ appointing and over appointing directors can significantly make SOEs augment their investment in innovation. Hence, H1 was confirmed.

**Table 3:** The regression results of power of non-state-owned shareholders in the board to SOE innovation investment.

Variable	Rd	Rd
	(1)	(2)
N_directorratio	0.0001***	
	(5.4599)	
N_gap		0.0001***
		(6.3098)
Constant	0.0911***	0.0895***
	(12.5423)	(12.3008)
Year & Ind	Controlled	Controlled
N	7746	7746
R^2^	0.387	0.387
adj. R^2^	0.383	0.383

Note: Values in parentheses are t-values; * p < 0.1, ** p < 0.05, *** p < 0.01, the same below.

#### The mediating role of board meeting frequency.

This study built models (1) to (4) to test the mediating role of board meeting frequency Table 4 reports the regression results of board meeting frequency serving as a mediator. Columns (1) and (3) show that the regression coefficients of the ratio of appointing directors by non-state-owned shareholders (N_directorratio) are positive at the 1% significance level; the regression coefficient for the ratio of directors appointed in excess by non-state-owned shareholders (N_gap) is −0.0121, which is negative at the 1% significance level. Columns (2) and (4) show that the regression coefficients of the two alternative variables of the power of non-state-owned shareholders in the board and board meeting frequency (Meeting) are all significantly positive. The results show that non-state appointed directors can promote innovation investment by increasing board meeting frequency. Hence, H2 was confirmed.

**Table 4:** The regression results of the mediating effect of board interplay intensity.

Variable	Meeting	Rd	Meeting	Rd
	(1)	(2)	(3)	(4)
Meeting		0.0001*		0.0002**
		(1.6659)		(2.0860)
N_director ratio	0.0079**	0.0001***		
	(2.4218)	(5.4125)		
N_gap			−0.0121***	0.0001***
			(−3.7131)	(6.3937)
Constant	−1.8638*	0.0913***	−0.8529	0.0896***
	(−1.7577)	(12.5746)	(−0.8029)	(12.3221)
Year & Ind	Controlled	Controlled	Controlled	Controlled
N	7746	7746	7746	7746
R^2^	0.136	0.387	0.136	0.388
adj. R^2^	0.130	0.383	0.131	0.384

#### The moderating role of the power of state-owned shareholders in the board.

This study set up Model (4) to test and prove the moderating effect of the power of state-owned shareholders in the board by creating interaction terms between two alternative variables of the power of non-state-owned shareholders in the board and the ratio of directors appointed by state-owned shareholders, as well as the squared term of the ratio of directors appointed by state-owned shareholders. To test the accuracy of the results, this paper conducted mean centering on the variables. As the regression coefficients of the interaction terms in Table 5 show, the coefficient of the power of non-state-owned shareholders in the board and that of state-owned shareholders (S_directorratio) are significant at the 1% level; the coefficients of the squared term between the power of non-state-owned shareholders in the board and that of state-owned shareholder (S_directorratio^2^) are significantly negative. Drawing on the explanations of the testing method for the curvilinear effect by Lin and Feng [76] and the research by Wen and Zhou [77], when the regression coefficient of the interaction term between the squared term of the power of state-owned shareholders in the board and the two alternative variables of the power of non-state-owned shareholders in the board is significantly negative, the power of state-owned shareholders in the board plays an inverted U-shaped role. In the positive relationship between the power of non-state shareholders in the board and the innovation investment of state-owned enterprises, as the power of state-owned shareholders in the board increases, the significant promotion effect on SOEs’ innovation investment is first strengthened and then weakened. Hence, H3 was confirmed.

**Table 5:** The regression results of the moderating role of the power of state-owned shareholders in the board.

Variable	Rd	Rd	Rd	Rd
	(1)	(2)	(3)	(4)
N_directorratio*S_directorratio		0.0000***		
		(4.8210)		
N_gap* S_directorratio				0.0000***
				(3.3667)
N_directorratio*S_directorratio^2^		−0.0000***		
		(−3.3422)		
N_gap* S_directorratio^2^				−0.0000**
				(−2.0322)
Constant	0.0909***	0.0897***	0.0902***	0.0867***
	(12.4336)	(12.3050)	(12.3801)	(11.8556)
Year & Ind	Controlled	Controlled	Controlled	Controlled
N	7746	7746	7746	7746
R^2^	0.388	0.390	0.388	0.389
adj. R^2^	0.383	0.386	0.384	0.385

### Robustness tests

#### Robustness test for replacement of independent variables.

First, the measurement method of independent variables is replaced by dummy variables, referring to Zheng [46] if there are non-state-owned directors on the board of directors of state-owned enterprises, the value is 1, otherwise it is 0, and if there are non-state-owned shareholders over-appointing directors to the board of directors, the value is 1, otherwise it is 0.

Moreover, in realities, apart from the board of directors, the board of supervisors also shoulders major supervisory responsibilities. Senior executives have relatively larger decision-making power over regular events. With reference to Li and Li [33], this paper treated whether non-state-owned shareholders appoint directors, supervisors and senior executives (N_djg) and the ratio of non-state-owned shareholders’ appointing directors, supervisors, senior executives (N_djgratio) as another two alternative variables, and on this basis re-conducted regression on the main hypotheses. The results further proved the promotion effect of the power of non-state-owned shareholder in the board on the innovation investment of SOEs, and confirmed the robustness of the research conclusions of this paper (Owing to space limit, robustness tests and further analysis results are not reported here. They are available upon request.).

#### Replacement of dependent variables.

In order to make the research conclusions of this paper more robust, by referring to the studies by Li et al. [28] and Boubakri et al. [78], the authors of this paper went further to use the ratio of company R&D investment to total assets as another alternative variable for innovation investment, and re-conducted regression on Model (1). The results show that the research conclusions of this paper are robust

#### Test for the lagged nature of innovation.

According to the pathway of Board Power – Board Function – Enterprise Innovation, the affecting of the power of non-state-owned shareholders in the board on enterprises’ innovation investment is a process, which is subject to numerous factors. Considering the lagged nature of innovation investment and referring to the studies by Li and Li [33] and Li et al. [28], the authors of this paper treated the innovation investment in the t + 1th year as the dependent variable and conducted a relevant robustness test. The results show that the regression coefficients of the two alternative variables of the power of non-state-owned shareholders in the board to the innovation investment of state-owned enterprises are significant at the 1% level. It indicates that the impacts of the power of non-state-owned shareholders in the board on SOEs’ innovation investment are indeed lagged to a certain extent, further testing and verifying the results of the main regression effect.

#### Test for propensity score matching (PSM).

Given the impacts of observable and non-observable factors, the test may be subject to the problems of sample self-selection and omitted variables. As a solution, by referring to Li and Li [33] and Li et al. [28], the authors of this paper adopted the propensity score method (PSM) to conduct a robustness test. The authors of this paper employed the nearest neighbor matching method to conduct 1:1 pairing, and used the samples that have passed the balance test to further re-run regression analysis on Model (1). The results of the PSM test further proved that the research conclusions of this paper are robust.

### Further analysis

#### Pooled testing for SOE hierarchical heterogeneity.

As SOEs’ hierarchy differs, SOEs’ positioning in national economy and their policy perception and burden also differ [79]. Based on the hierarchical category of SOEs, the authors of this paper employed the pooled testing method to analyze the heterogeneous impacts of the power of non-state-owned shareholders in the board on innovation investment. The results show that in the samples of local SOEs, the regression coefficients of the four alternative variables of the power of non-state-owned shareholders in the board to SOEs’ innovation investment are higher than those of the samples of central state-owned enterprises. One possible explanation is that, central state-owned enterprises, as an important component of SOEs, are under direct governance of the State Council, and are to solve fundamental problems important to people’s well-being. In that case, non-state-owned directors only have relatively limited impacts on the decision-making concerning the innovation by central state-owned enterprises, and consequently cannot bring the governance effect of non-state-owned shareholders into full play. Local officers, who are strongly motivated by political promotion, are more likely to intervene in the behaviors of local SOEs [21]. Specifically, they intervene in the behaviors of state-owned directors by pressing ahead with reforms. In that case, the participation of non-state-owned shareholders in governance has more profound influence upon the supervision and decision-making of the board. In summary, to local SOEs, the power of non-state-owned shareholders in the board can better promote SOEs’ innovation investment.

### Pooled testing for the industrial heterogeneity of SOEs

#### Pooled testing for whether an enterprise is a high-tech enterprise.

High-tech enterprises refer to the enterprises that are committed to innovation research and development and innovation achievement transformation in areas supported with prioritized state policies. Compared to non-high-tech enterprises, high-tech enterprises are more acute of and self-driven for innovation. Drawing on Peng and Mao [80], the authors of this paper categorized SOEs into high-tech enterprises and non-high-tech enterprises, and on this basis conducted pooled testing for their industrial heterogeneity. The results show that in the sub-samples of high-tech enterprises, the coefficients of the two alternative variables of the power of non-state-owned shareholders in the board to SOEs’ innovation investment are all larger than those in the sub-samples of non-high-tech enterprises. Although high-tech enterprises have a strong desire for innovation, the advance of their innovation is hampered due to governmental intervention and rigid systems. In summary, for high-tech enterprises, the power of non-state-owned shareholders in the board has a more significant promotion effect on SOEs’ innovation investment.

#### Pooled testing for whether an enterprise is a SOE for commercial priority.

SOEs undertake differentiated social functions as their types vary. SOEs can be put into three categories, namely, SOEs for commercial priority, for special functions, and for public services [81]. In accordance with the standards for the classification of SOEs by Wei et al. [82], the authors of this paper screened out SOEs for commercial priority, and conducted pooled testing for the heterogeneity of SOE types. Among the sub-samples of SOEs for commercial priority, the coefficients of the two virtual alternative variables of the power of non-state-owned shareholders in the board to SOEs’ innovation investment are both smaller than those in the sub-samples of SOEs for non-commercial priority. The coefficients of the two continuous alternative variables of the power of non-state-owned shareholders in the board to SOE innovation investment are both larger than those in the sub-samples of SOEs for non-commercial priority. Since continuous variables have a stronger capacity of explanation, it indicates that among SOEs for commercial priority, the power of non-state-owned shareholders in the board has greater influence on SOEs’ innovation investment. SOEs for commercial priority place themselves in competitive industries and aim at seeking profits. In theory, they and non-state-owned enterprises share the same market positioning and goals [28]. SOEs for commercial priority are more motivated for innovation investment and call for reforms to turn around the situation featuring low innovation level and the lack of sustainable market competitiveness.

## Conclusions and implications

### Research conclusions

First, the power of non-state-owned shareholders in the board has a significant positive impact on SOEs’ investment in innovation. The results of the robustness test show that such a finding holds. Concretely, both non-state-owned shareholders’ appointing and over appointing directors can significantly drive SOEs to amplify their investment in innovation. Regarding the research on the participation of non-state-owned shareholders in corporate governance, few existing studies focus on board power. In this paper, the power of non-state-owned shareholders in the board was defined. The principal-agent theory, the theory of resource dependence and the no-equal allocation logic between equity and control right were employed to make a systematic analysis of the theoretical and practical impacts of the power of non-state-owned shareholders in the board. Such findings add valuable assets to the extant literature on the theoretical analysis and practical tests of the power of non-state-owned shareholders in the board.

Second, the interplay intensity in the board plays a partial mediating role in the impact of the power of non-state-owned shareholders in the board on the innovation investment of state-owned enterprises. When non-state-owned shareholders obtain power from the board, the board’s functions of supervision and decision-making can be better performed. Specifically speaking, the intensity of interplay in the board is reinforced. With the entry of non-state-owned directors into the SOE board, the interplay in the board is intensified, that is, the communication between members of the board is increased. The supervision effect of non-state-owned directors is strengthened, and the quality of decision-making of the board is improved. This can guarantee the supply of resources essential for innovation investment, and advance the level of SOE innovation investment.

Third, the power of state-owned shareholders in the board plays an inverted U-shaped role in the positive promotion effect exerted by the power of non-state-owned shareholders in the board on SOE’s investment in innovation. When the power of state-owned shareholders in the board is confined to a certain scope, it will fortify the promotion effect of the power of non-state-owned shareholders in the board on SOEs’ innovation investment. When the power of state-owned shareholders in the board is beyond a certain critical point, it will inhibit the promotion effect of the power of non-state-owned shareholders in the board on SOE innovation investment.

### Managerial implications

First, the steady advance of the mixed ownership reform of SOEs entails a focus on the power allocation of the board

After SOEs introduce non-state-owned capital, how to effectively allocate the power of the board is a priority and a challenge. If SOEs desire to go beyond capital mixing and pursue a mechanism reform, such a challenge must be overcome. The conclusions of this paper indicate that the power of non-state-owned shareholders in the board can effectively promote the innovation capacity of SOEs. In summary, introducing non-state-owned capital is far from being enough for the reform of SOEs. It is proposed that an appropriate or even excess number of non-state-owned directors be brought in to the board, and certain say should be given to non-state-owned shareholders in the board. It will help supervise state-owned shareholders and senior executives more effectively, and change the arbitrary situation of the state-owned board. Central and local governments should delegate their power, and provide sound policy guidance for non-state-owned shareholders to enter the state-owned board and engage in relevant governance and even obtain excess power in the board.

Second, efforts should be made to guard against extreme governmental power delegation to prevent unnecessary counteraction.

It is essential that non-state-owned shareholders obtain excess power in the board to substantially offset the dominance of state-owned shareholders in the board. However, blind power delegation may alter the situation between state-owned directors and non-state-owned directors. The state-owned directors who should be supervised may end up checking and balancing against non-state-owned directors who possess too much power. In that case, the board will become the battle ground for power between state-owned directors and non-state-owned directors, with neither side making sound and scientific decisions for the management of the enterprise. In summary, on the matter of board power allocation, due regard should be given to the sizes of both state-owned and non-state-owned parties so as to propel the SOE mixed ownership reform to achieve the desired results.

Third, at the enterprise level, a scientific and effective board should be developed by giving priority to the effective execution of the board’s functions.

The board system is an important component of the modern enterprise system with distinct Chinese features. To develop and improve the board system is a major task facing the SOE reform. Based on the two functions of the board, namely, supervision and decision-making, this paper analyzed, tested and proved the mediating role of the interplay intensity in the board in the process when non-state-owned shareholders exert impacts on SOEs’ innovation investment. Following the introduction of non-state-owned directors, SOEs should make full use of the heterogeneous resources brought by non-state-owned directors so as to foster sound interplay between state-owned directors and non-state-owned directors. Mutual consultation, cooperation and negotiation should be undertaken to improve the decision-making and supervision functions of the board, develop a better SOE board system, so as to achieve long-term market competitiveness with concerted efforts.

### Limitations and future research

First, in terms of variable choosing, the measuring of the power of non-state-owned shareholders in the board remains to be further discussed.

The number of seats of directors in the board is a straightforward index showing that non-state-owned shareholders obtain a say in the board. According to the extant research, the director who assumes the post of director in a SOE and simultaneously takes a post in a non-SOE and receives a salary from the non-SOE instead of the SOE is regarded as a director appointed by the non-SOE. However, such a measuring method has its own limitations. For one thing, the annual report of a listed SOE may offer incomplete disclosure of whether its directors assume a post in a non-SOE and whether the relevant directors receive salaries from the non-SOE. For another thing, under the influence of distinct governance culture in SOEs, such as the strict observance of status and order, the clear line between the insider and the outsider [57], it remains a question whether the size of the power of non-state-owned shareholders in the board can be directly measured by the ratio of the seats of non-state-owned shareholders in the board. In addition, for the lack of data, this study has not yet been able to further explore the impacts of the commissions of audit, strategy, nomination, remuneration, and other commissions under the board of directors on the substantial power of non-state-owned shareholders in the board. This is also one of the directions worth studying in the future.

Second, the discussions of the action mechanism are not thorough enough.

Taking the effectiveness of board governance as the starting point, this paper studied the promotion effect of the power of non-state-owned shareholders in the board on the functional performance of the board and its corresponding impacts on the innovation investment of SOEs. The discussions of the mechanism remain to be improved in the following aspects. Extant studies mainly consider board structure, diversity, diligence while neglecting board functions. In the studies by Gong and Mao [83,84], the performance of the board’s supervision function is measured by accounting information quality and senior executives’ excess salary, and the performance of the board’s consultation function is measured by innovation initiatives and operational performance. However, these indicators do not directly or accurately present the functions of the board. There are multiple paths available for the power of non-state-owned shareholders in the board to affect SOEs’ innovation investment. Follow-up studies can make further exploration in fields such as the role of non-formal level of the board in SOEs’ innovation and the impacts of external governance on SOEs’ innovation investment.

Third, this paper has not yet considered the dynamic relationship between shareholders’ power, heterogeneity and board power, that is, in the course of the mixed ownership reform of SOEs, how state-owned equity and non-state-owned equity affect the power allocation of the board and even the management, and how to allocate power under the dominance of different types of equity. The important goal of the mixed ownership reform of SOEs is to advance SOE innovation so as to promote SOE high-quality development. However, the course evolving from shareholder capital mixing to board mechanism reform, and to enterprise innovation is mixed with complex logic relationships. How capital mixing affects power allocation and how board power can be dynamically adjusted are issues to be further explored in the studies on SOE innovation.

Forth, although this paper employs lagged models and PSM to mitigate endogeneity concerns, it cannot fully rule out biases arising from unobservable factors or omitted variables. Future research could focus on identifying more effective instrumental variables or leveraging exogenous policy shocks to construct quasi-natural experiments, such as pilot policies for mixed-ownership reform, thereby further verifying the causality of the findings presented here.
